# CD133: Enhancement of Bone Healing by Local Transplantation of Peripheral Blood Cells in a Biologically Delayed Rat Osteotomy Model

**DOI:** 10.1371/journal.pone.0052650

**Published:** 2013-02-14

**Authors:** Bernd Preininger, Georg Duda, Hinnerk Gerigk, Jonas Bruckner, Agnes Ellinghaus, F. Andrea Sass, Carsten Perka, Katharina Schmidt-Bleek, Anke Dienelt

**Affiliations:** 1 Julius Wolff Institut and Center for Musculoskeletal Surgery, Charité – University Medicine Berlin, Berlin, Germany; 2 Orthopaedic Department, Center for Musculoskeletal Surgery, Charité – University Medicine Berlin, Berlin, Germany; 3 Berlin-Brandenburg Center for Regenerative Therapies, Charité – University Medicine Berlin, Berlin, Germany; Leibniz Institute of Age Research – Fritz Lipmann Institute, Germany

## Abstract

Sufficient angiogenesis is crucial during tissue regeneration and therefore also pivotal in bone defect healing. Recently, peripheral blood derived progenitor cells have been identified to have in addition to their angiogenic potential also osteogenic characteristics, leading to the hypothesis that bone regeneration could be stimulated by local administration of these cells. The aim of this study was to evaluate the angiogenic potential of locally administered progenitor cells to improve bone defect healing. Cells were separated from the peripheral blood of donor animals using the markers CD34 and CD133. Results of the *in vitro* experiments confirmed high angiogenic potential in the CD133(+) cell group. CD34(+) and CD133(+) cells were tested in an in vivo rat femoral defect model of delayed healing for their positive effect on the healing outcome. An increased callus formation and higher bone mineral density of callus tissue was found after the CD133(+) cell treatment compared to the group treated with CD34(+) cells and the control group without cells. Histological findings confirmed an increase in vessel formation and mineralization at day 42 in the osteotomy gap after CD133(+) cell transplantation. The higher angiogenic potential of CD133(+) cells from the *in vitro* experients therefore correlates with the *in vivo* data. This study demonstrates the suitability of angiogenic precursors to further bone healing and gives an indication that peripheral blood is a promising source for progenitor cells circumventing the problems associated with bone marrow extraction.

## Introduction

Tissue regeneration closely depends on the vascular supply and deficient healing situations are frequently associated with poor blood perfusion. Such a vascular supply is crucial in the treatment of segmental bone defects or spinal fusions where poor healing outcomes as delayed unions or pseudarthrosis are frequently associated with poor angiogenesis [1], [2]. It has been demonstrated in animal models that an inhibition of angiogenesis leads to a defective bone healing [3]. Thus, it can be assumed that bone healing could be improved by stimulating angiogenesis and vessel formation at the site of regeneration, providing the basic prerequisite for possible regenerative processes [4].

Mesenchymal stromal cells (MSCs) are able to differentiate into several cell types of the mesodermal lineage, such as osteoblast or chondrocytes [5]. Additionally these cells have pro-angiogenic properties and facilitate the formation of new blood vessels by secretion of factors such as VEGF-A (vascular endothelial growth factor A), b-FGF (basic fibroblast growth factor), and IGF-1 (insulin-like growth factor-1, erythropoietic factor) [6], [7]. In accordance with their high regenerative capacity transplanted MSCs improve fracture healing as was shown in different animal studies [8], [9]. A limitation to transfer this application into a broader setup for the therapeutic use in the clinic is the time and cost intensive isolation of MSCs by bone marrow aspiration, together with an *ex vivo* expansion that is necessary to reach a certain number of cells [10]. On top, the harvesting of bone marrow makes an earlier intervention prior the fracture treatment necessary that is associated with additional morbidities which ideally should be avoided. An alternative to the application of MSCs would be the use of a tissue source containing a sufficient amount of progenitor cells that can be obtained easily with low donor side morbidity but represent a similar pro-angiogenic potential as described for MSCs. Peripheral blood seems to be a good option for this strategy.

Several progenitor cell populations isolated from bone marrow that were reported to have angiogenic properties are also found circulating in the peripheral blood. CD34(+) cells are able to differentiate into endothelial cells [11] and can possibly support the formation of new blood vessels in vivo [12], [13]. Other cells that were shown to support angiogenesis in experimental in vivo models are CD133(+) cells [14]. CD133(+) cells are ancestral precursors of CD34(+) cells [15] with angiogenic potential [14], [16], [17].

The aim of this study was to investigate whether a sufficiently pro-angiogenic cell source could be identified from peripheral blood that would allow stimulating tissue regeneration such as in bone defect healing under impaired conditions. The regenerative potential of the cells with the high angiogenic capacity were subsequently analyzed towards their ability to enhance the healing outcome in a rat osteotomy model of impaired bone healing. The results gained from this study can serve to evaluate the local administration of angiogenic precursors cells in future treatment strategies for patients suffering from impaired bone healing situations.

## Materials and Methods

### *In vitro* characterization: flow cytometric analysis

1×10^6^ PBMCs were resuspended in FC-buffer (DPBS supplemented with 1% bovine serum albumin and 0.1% NaN_3_) and stained with CD133-2-PE antibody (Miltenyi Biotec GmbH, Bergisch Gladbach, Germany), CD14-VioBlue (Miltenyi Biotec GmbH, Bergisch Gladbach, Germany) antibody and CD34-APC (Sigma-Aldrich, Munich, Germany) after blocking of unspecific binding sites by addition of FcR solution and bovine serum albumin. Stained cells were washed twice with FC-buffer, counterstained with 4′,6-diamidino-2-phenylindole (DAPI) and subsequently analyzed for the occurrence of viable CD34+/CD133+ lymphocytes at a MACSQuant flow cytometer (Miltenyi Biotec GmbH, Bergisch Gladbach, Germany). For the identification of viable lymphocytes a forward/sideward scatter gate containing all lymphocytes and monocytes was set. Subsequently dead cells stained with DAPI and monocytes stained positive for CD14 were excluded via fluorescence measurements.

### *In vitro* characterization: Tube formation Assay

Peripheral blood mononuclear cells (PBMCs) were isolated from healthy human peripheral blood derived Buffy Coats (provided from DRK, (Deutsches Rotes Kreuz) by Histopaque-1077 (Sigma-Aldrich, Munich, Germany) density gradient centrifugation. Therefore the blood was diluted with Dulbecco's phosphate buffered saline (DPBS) at a ratio 1∶1. 25 ml blood/DPBS solution were loaded onto 15 ml Histopaque, centrifuged at 400 g for 30 min without brake. The PBMCs containing layer was aspirated, transferred into a new tube and washed twice with DPBS/2mM EDTA. CD34 and CD133 positive and negative cell subpopulations were isolated from total PBMCs by immunomagnetic cell separation (MACS System; Miltenyi Biotec, Bergisch Gladbach, Germany) according to the manufacture's instruction with the provided respective antibody. Human umbilical vein endothelial cells (HUVECs) were obtained from Lonza (Lonza Cologne AG, Cologne, Germany) and grown in Endothelial Growth Medium supplemented with growth factors (EGM and EGM-BulletKit, Lonza Cologne AG, Cologne, Germany), 100 U/mL penicillin, 100 μg/mL streptomycin and 2% fetal calf serum (FCS).

5×10^4^ HUVECs/well were plated onto 24well plates coated with 50 µl growth factor reduced Matrigel (BD Biosciences, Heidelberg, Germany). 5×10^4^ magnetically sorted cell subpopulations were seeded into cell culture inserts (1.0 µm, BD Biosciences Falcon, Bioscience, Heidelberg, Germany) and cultivated together with the HUVECs for 18 hours in EGM medium without growth factors, supplemented with 100 U/ml penicillin, 100 µg/ml streptomycin and 2% FCS. Thus the two cell types shared the culture medium but were separated by the membrane of the insert. Tube formation of the HUVECs was documented after 18 hours of co-culture by bright field microscopy. The total length of tubes formed in each well was measured by ImageJ (version 1.44p; http://rsbweb.nih.gov/ij/) and correlated to the tube length formed in HUVEC co-cultures with unsorted PBMCs that served as internal control.

### *In vitro* characterization: qPCR gene expression analysis

Total RNA was isolated from PBMCs, CD34(+)/(−) and CD133(+)/(−) cells by Trizol extraction according to the manufactors instruction (Life Technologies GmbH, Darmstadt, Germany) [18]. The RNA concentration was determined with a Nano-Drop spectrophotometer by measuring the absorbance at 260 and 280 nm. Samples with an inappropriate 260/280 ratio were excluded from further analysis. For quantitative PCR, cDNA was synthesized from 250 ng RNA in a total volume of 25 µL with iScript reverse transcriptase as indicated by the manufacturer (Bio-Rad Laboratories GmbH, Munich, Germany). Primer sequences were generated with primer 3 (http://frodo.wi.mit.edu/) and tested for specificity with reverse ePCR (http://www.ncbi.nlm.nih.gov/projects/e-pcr/). The following primer sequences were used for analysis of the expression of basic fibroblast growth factor (b-FGF), vascular endothelial growth factor a (VEGFa), platelet derived growth factor a (PDGFa) and tata-box binding protein (Tbp): b-FGF-for 5′-agcggctgtactgcaaaaac-3′, b-FGF-rev 5′-cttgatgtgagggtcgctct-3′, VEGFa-for 5′-cccactgaggagtccaacat-3′, VEGFa-rev 5′-cctcggcttgtcacattttt-3′, PDGFa-for 5′-caagaccaggacggtcattt-3′, PDGFa-rev 5′-cttgacactgctcgtgttgc-3′, Tbp-for 5′-tataatcccaagcggtttgc-3′, Tbp-rev 5′-gcacaccattttcccagaac-3′. Using an iQ5 Cycler (Bio-Rad Laboratories GmbH, Munich, Germany) 1 µl of the first strand reaction was analyzed for quantitative gene expression in a SYBR Green PCR master mix (Bio-Rad Laboratories GmbH, Munich, Germany) with cycle conditions as quoted before [19]. Expression of each gene was calculated according to the ddCT method as reported by Pfaffl with adjustment for PCR efficiency [20]. The expression was normalized to the expression of the TATA-box binding protein (Tbp) (the housekeeping gene was tested against others and was fount do be the most stable with the investigated samples) and compared to the expression seen in analysis of total PBMCs.

### *In vitro* preparation: Grafts to locally administer pluripotent cells

Whole blood was drawn from donor animals (female ex-breeder Sprague-Dawley rats, minimum 3 litters, age 12 months). CD34(+) and CD133(+) cells were separated by Histopaque-1083 (Sigma-Aldrich, Munich, Germany) density centrifugation and isolated subsequently following the MACS Technology protocol established by Miltenyi Biotech (Miltenyi Biotec GmbH, Bergisch Gladbach, Germany) using biotinylated CD34 (Miltenyl #130-046-702, Miltenyi Biotec, Bergisch Gladbach, Germany) or CD133 antibody (Ab16518-100 abcam lot:746560, abcam, Cambridge, UK) and anti-biotin microbeads. 2×10^5^ CD34(+) or CD133(+) cells were resuspended afterwards in 200 µl autologous whole blood. Immediately thereafter a blood clot was prepared. From each animal 200 μl of autologous peripheral blood were drawn from the right saphenic vein directly prior to surgery. Immediate coagulation was inhibited by addition of 30 µl citrate (buffered sodium citrate (equiv. to 3.2% sodium citrate), taken from BD vacutainer citrate plus plastic tubes (#363080, BD Franklin Lakes, NJ, USA). The blood was then filled into the lid of a 1.5 ml Eppendorf tube (VER International GmbH, Darmstadt, Germany) as a form giving device for the round shape of the graft to fit into the osteotomy gap. In the cell-groups (CD133(+) and CD34(+)) 2×10^5^ cells were added before clotting was initiated with addition of 4 µl CaCl_2_ 12% and 4 µl Thrombin (500 I.E./mL; Baxter Deutschland GmbH, Unterschleißheim, Germany). In the control group (ABC) the autologous clot was transplanted without addition of isolated cells.

### *In vivo* Experimental Design: Bone defect healing

The capacity of the angiogenic potential of progenitor cells was evaluated in a small animal model for impaired bone healing. The model accounted for major risk factors such as age, female sex, and the number of parities [21]–[28]. In 19 female ex breeder Sprague-Dawley rats (age 12 months, weight: 412 g ±22; minimum 3 litters) a mid-femoral double osteotomy was stabilized with an external fixator [21], [29]. The double osteotomy led to a 2 mm gap between the cortices. In one group a bare artificial blood clot was implanted into the gap (ABC, n = 6), in the second group a CD133(+) cell-charged blood clot was implanted into the gap (CD 133(+), n = 6) and in a third group a CD34(+) cell-charged blood clot was added (CD34(+), n = 7). The data of 5 of the animals of the ABC-group has been used in a previous publication [21]. All animal experiments were carried out according to the policies and principles established by the AnimalWelfare Act. The design of the animal surgeries was critically reviewed and approved by the local legal representative (Landesamt für Arbeitsschutz, Gesundheitsschutz und technische Sicherheit, Berlin: G 0428/08).

### Surgical technique

The exact surgical procedure has been described before [21]. In brief: Under general intraperitoneally applied anaesthesia. (0.3 mg/kg Medetomidin Domitor®, Pfizer, Karlsruhe, Germany) and 60 mg/kg Ketamin (Actavis, Langenfeld, Germany) the left hind portion, including the entire leg, was shaved and disinfected by using povidone-iodine. An antibiotic (45 mg/kg Clindamycin, Ratiopharm, Ulm, Germany) was subcutaneously injected. For analgesia prior to the surgery the animals received 20 mg/kg Tramadol (Tramal®, Grünendal Aachen, Germany). A longitudinal lateral approach was made over the femur. A drilling guide to ensured correct and reproducible positioning of the K-wires was used to consecutively drill four holes using constant irrigation (0.9% saline solution) and followed by screwing in the wires bicortically. The cross-linked carbon fixator bar was mounted on the wires at an offset of 7.5 mm and the femur was double osteotomized (0.3-mm saw blade, S-8R, Implantmeds, W&H Oral Surgery, Bürmoos, Austria) in the centre between the two inner K-wires before the bone segment was removed.

Into that osteotomy gap either the bare (ABC), the CD34(+) or the CD133 (+) charged artificial blood clot was positioned. The fascia of the muscle was sutured with 3.0 absorbable sutures, the skin then closed with 3.0 non-absorbable sutures, which were removed after 2 weeks during the wound care routine. Post-surgery analgesia was administered through the drinking water (Tramal® (25 ml/l) for three days).

### *In vivo* Micro Computer Tomography (µCT)

After surgery bone healing was assessed by *in vivo* µCT at 2, 4, and 6 weeks under general anaesthesia (Medetomidin 0.3 mg/kg Domitor®, Pfizer, Karlsruhe, Gemany) and Ketamin 60 mg/kg (Actavis, Langenfeld, Germany) i.p.). Quantitative micro-computed tomography (vivaCT 40, Scanco, Switzerland) was applied to scan all femora and reconstruction was performed at 35 μm isotropic resolution. A volume of interest (VOI) was defined for the periosteal and endosteal callus, excluding the cortical bone and an axially 4 mm wide VOI centered to the OT-gap was applied.

A global threshold for fracture callus at 233 mg HA/cm3 was set, corresponding to half of the mineralization of intact tibial cortex based on visual inspection of ten random single tomographic tibial slices from each animal. Readout parameters were bone volume (BV, mm3), total callus volume (CV, mm3), BV/CV, and tissue mineral density (TMD, mg HA/cm3). Bone mineral content (BMC) was calculated as a product of BV and BMD. 3D Pictures were reconstructed using Scanco software [30].

Bridging of the OT gap by mineralized tissue was quantified by a bridging score described earlier [21]. In brief: The OT Gap was divided into 5 regions and mineralized tissue formations bridging the gap were assessed in those 5 separate regions (12, 3, 6, 9 o'clock and centre). One blinded scorer evaluated bridging and every separate region, bridging was rated 1 for bridged or 0 for non-bridged. The bridging score for every individual animal represents the sum of the scores of every region with a maximum of 5 points.

### Histological Procedure and Analyses

The femurs were fixed for 2 days in normal buffered formaldehyde, decalcified in EDTA (14 days at 37°C), dehydrated with alcohol and xylol, and embedded in paraffin. Longitudinal serial sections (4 µm) were cut in the plane of the K-wire ducts.

Sections were stained with Movat's Pentachrome to allow distinction between the different tissue types (bone yellow, cartilage green, connective tissue red, nuclei black) [31]. Endothelial cells formed in capillaries and vessels were identified by immune staining for faktor VIII (vWF; von Willebrandt factor). The sections were analysed for the type of tissue bridging the gap. If gap bridging was reached, mineral tissue formation was differentiated into periosteal or intercortical regions. In addition, the number of capillaries and vessels formed was counted within a determined region of interest (ROI). The ROI has been previously defined 4 mm wide centered to the middle of the OT-gap containing the entire region of callus formation captured on the histological slice.

### Statistical Analyses

Mean values together with standard deviation were calculated for non-parametric data gained from the tube formation, qPCR analysis and µCT analysis. Data comparison was interpreted using the Man Whitney U Test. For statistical analysis SPSS 19.0 for Windows (SPSS Inc., Chicago, IL) was used. A p value of 0.05 or less was taken as a significant difference.

## Results

### Angiogenic capacity of peripheral blood derived CD34(+) and CD133(+) cells

As CD133(+) cells are described to be precursors of CD34(+) cells we first checked the analogy of these two cell populations. Indeed we found that there is a slight overlap of both cell population, but only a small number (8.34±4.52%) of CD34(+) lymphocytes express additional the marker CD133. Hence most cell express only one of the two markers CD34 or CD133 respectively and the function of both cell populations can be evaluated independently of each other.

**Figure 1:**
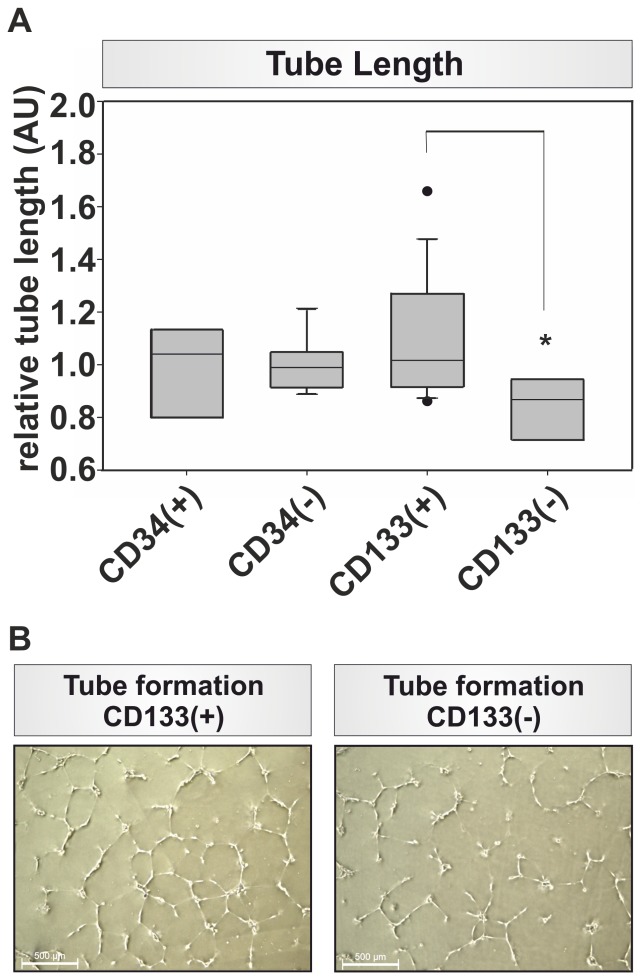
*In vitro* analysis of angiogenic potential of progenitor cells. (A) Tube length evaluation for CD34 (+)/(−) and CD133(+)/(−) cells normalized to the tube length measured in the control group together with all PBMCs, note the significant difference in tube length between CD133(+) and CD133(−), n = 8, * p = 0.018; (B) Tube formation of CD133(+) and CD133(−) respectively, note the more pronounced tube formation in the CD133(+) fraction.

Subsequently, we isolated CD34(+) and CD133(+) cells from PBMCs of whole blood and analyzed them for their angiogenic potential in tube formation assays. CD34(+) cells showed no significantly different impact on the tube length formed in comparison to the respective CD34(−) cell populations (0,973±0,192AU vs. 0,999±0,102AU; Fig. 1). In contrast, CD133(+) cells showed a 1.25 fold significant increase in tube length in comparison to the tube length formed in co-cultures with CD133(−) cells (1,088±0,288AU vs. 0,834±0,121AU; p = 0.0108; Fig. 1).

### Growth factor expression in CD34(+) and CD133(+) cells

**Figure 2:**
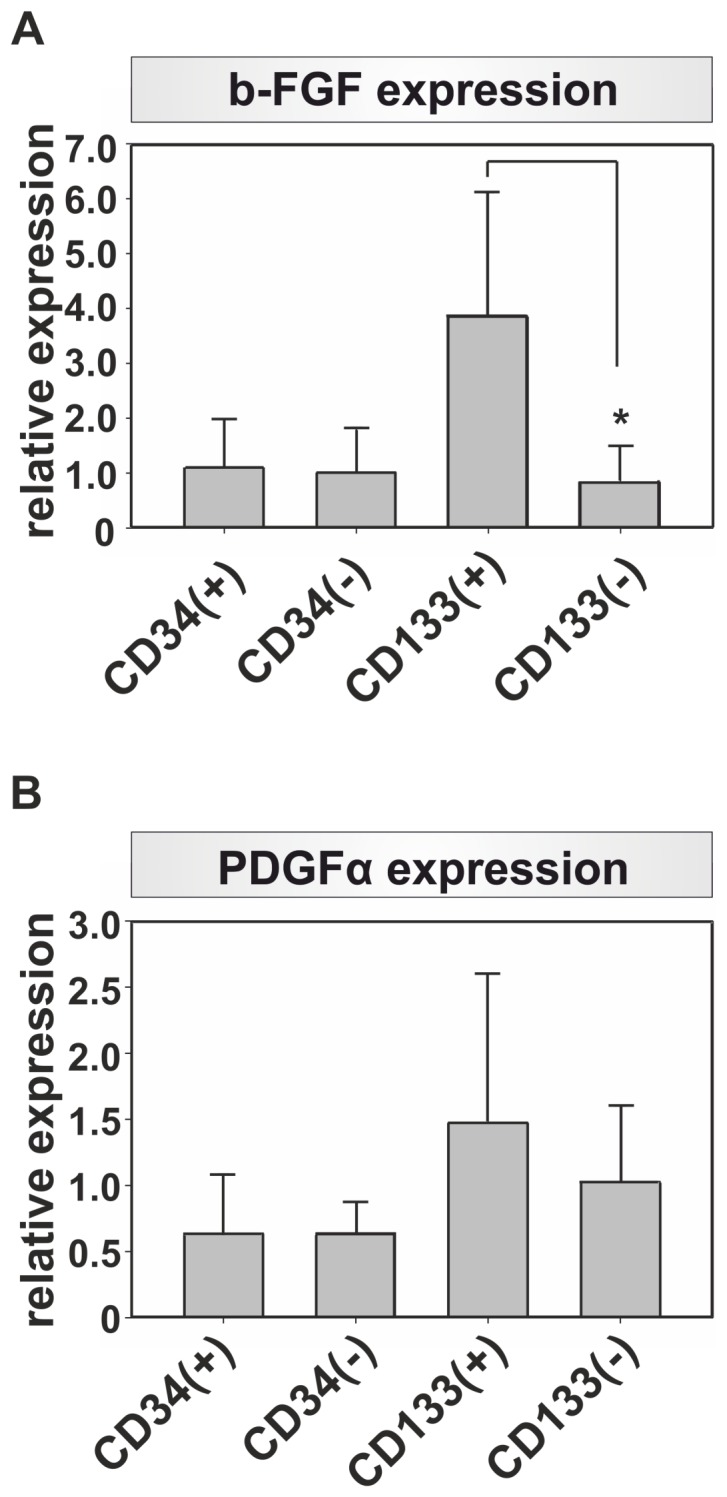
Growth factor gene expression analysis. The gene expression of (A) b-FGF, (B) PDGFa were analyzed with quantitative PCR and normlaized to the expression of Tbp. Note the more pronounced gene expression of b-FGF and PDGFa in CD133(+) cells, n = 5, * p = 0.009.

Beside their impact on tube formation, the expression of several pro-angiogenic growth factors was analyzed. We found that b-FGF and PDGFa were expressed 3.5fold and respectively 2.3fold more pronounced in the CD133(+) cell population in comparison to the CD34(+) cell population. However, due to the high standard deviations found in the gene expression of the different subpopulations, only the difference in the b-FGF expression in CD133(+) cells in comparison to the CD133(−) cells was found to be significantly altered (p = 0.009) (Fig. 2A/B).

### Bone formation: µCT revealed an earlier and pronounced bone formation in the CD133(+) group

To test the impact of peripheral blood derived cells on fracture repair, we analyzed the healing outcome after transplantation of CD34(+) and CD133(+) cells in a rat osteotomy model for impaired bone healing.

**Figure 3:**
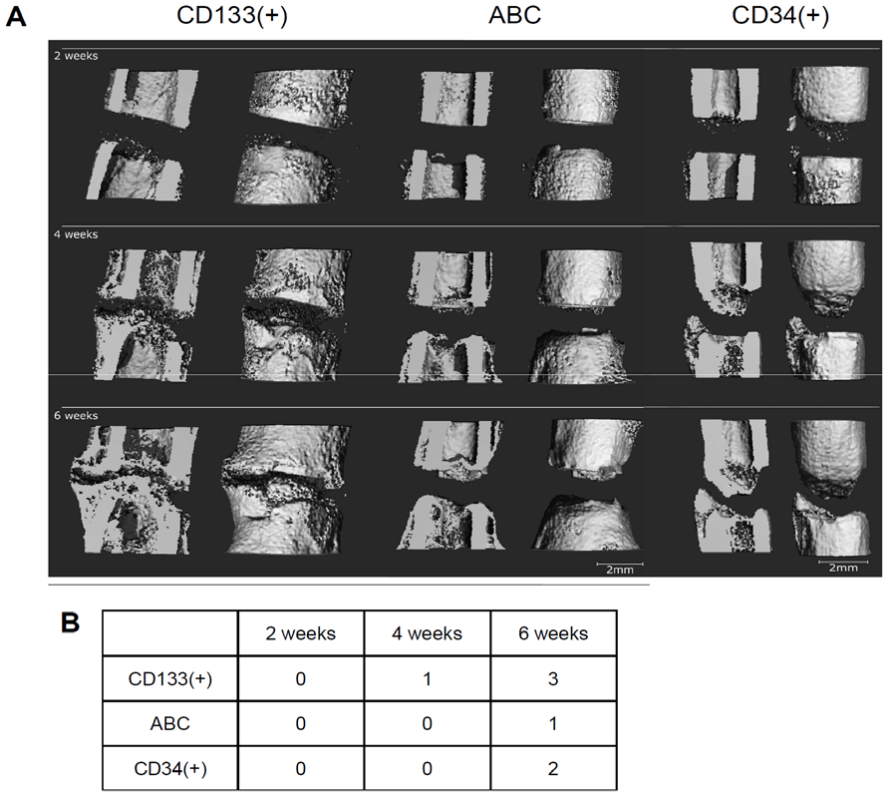
µCT analysis – 3D reconstructions of the bone healing progress. (**A**) 3D reconstructions from µCT scans from one representative animal after 2, 4, and 6 weeks of the three groups are shown. In the CD133(+) group (left) mineralized tissue was formed at the endosteal as well as at the periosteal regions. After 42 days the gap had almost been bridged; a thin layer of radiolucent tissue still separated the bone ends. In the ABC group (middle) only very little callus formation was observed, appearing between the 28th and 42nd day after the osteotomy. No bridging but a cap-formation was observed, sealing the medullary canal. In the CD34(+) group (right) bone formation was further progressed than in the ABC group. After 6 weeks however, bridging did not occur and the medullary canal was closed at the proximal end. (Resolution 28 µm; scale: 2 mm). (**B**) Bridging score for the ABC group is lower compared with the bridging in the CD34(+) or CD133(+) group. The bridging score for the CD34(+) group showed better results than the ABC group but lesser results compared with the CD133(+). 5 areas in the osteotomy gap were investigated, 12, 3, 6, and 9 o'clock position and the centre region. The sum of bridged areas for the healing course is indicated in the table.

Bridging of the osteotomy gap occurred earlier and was more pronounced in the CD133(+) group when compared with both the CD34(+) and the ABC group (Fig. 3).

**Figure 4:**
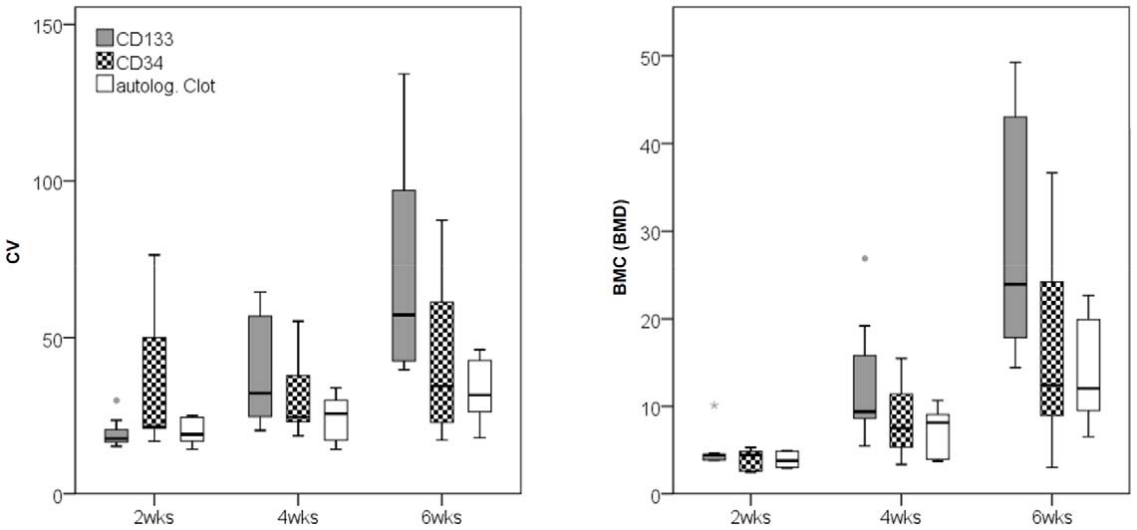
µCT analysis – statistic evaluation of callus volume and bone mineral contend. An increase of callus volume (CV, [mm3]), as well as bone mineral contend (BMC, [mg]) on the basis of total mineral density of the callus was observed over time. Both parameters were significantly higher in the CD133(+) group when compared to the ABC group. Note that the differences occur not until after the 28th day and are most pronounced after the 42nd day. Comparing CV and BMC between the CD133(+) group and the CD34(+) group markedly higher values were found in the CD133(+) group. (CV: total volume, BMC: bone mineral content, wks: weeks); (CD133(+) group  =  grey; CD34(+) group  =  checkered; ABC (autologous blood clot) group  =  white).

The volume of callus tissue (CV) formed in the osteotomy gap increased over time and was significantly higher in the CD133(+) group (p = 0.024) when compared with the ABC group (Fig. 4). For the CD34(+) cell group no significant increase in CV was observed when bone healing was compared to the ABC group (Fig. 4). The comparison between the CD133(+) group and the CD34(+) group revealed a better healing with higher CV values in the CD133(+) group (p = 0.061) (Fig. 4).

The BMC based on the BMD of the callus tissue formed increased over time and was significantly higher in the CD133(+) group (p = 0.022) when compared with the ABC group (Fig. 4). No significant increase in BMC was detected when comparing CD34(+) with the ABC group. The BMC was notably higher in the CD133(+) group when compared with the CD34(+) group (p = 0.051) (Fig. 4).

Both the amount and the grade of mineralization could be best increased by the addition of 2×10^5^ CD133(+) cells from the peripheral blood directly after the bone injury.

### Descriptive histology confirmed a higher mineralization in the CD133(+) cell treated group

To further analyze the impact of CD133(+) cells on bone healing, we executed histological analysis of the osteotomy site from animals treated with either the blood clot or the CD133(+) cells.

**Figure 5:**
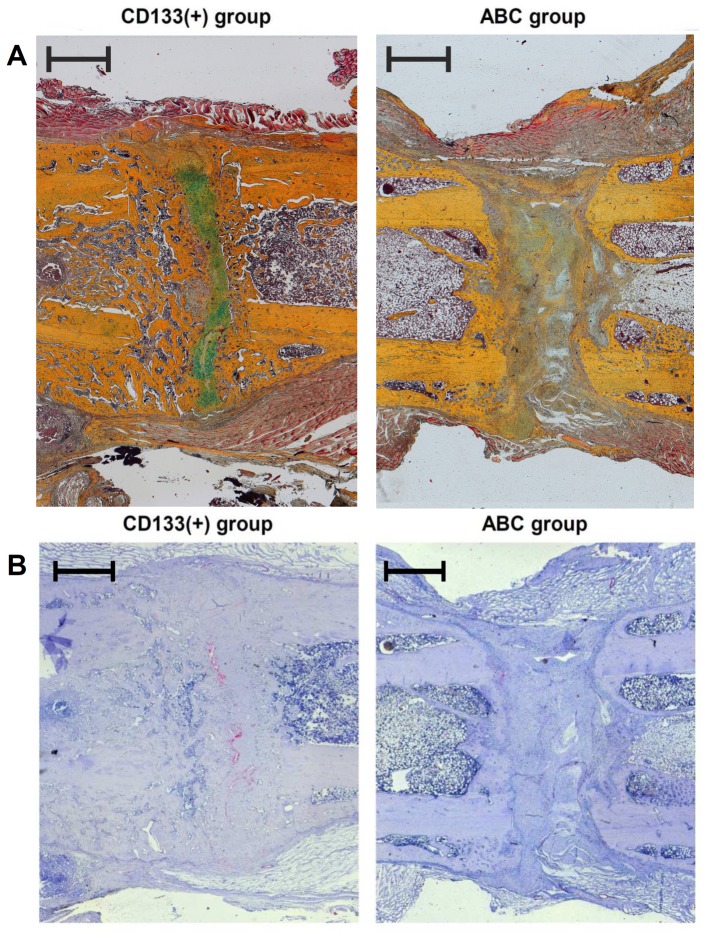
Histological analysis of bone formation and revascularization in the osteotomy gap. (**A**) Photomicrographs of histological sections 6 weeks post-surgery of the CD133(+) (left) and the ABC (right) group (Movat Pentachrome staining); In the CD133(+) group the osteotomy gap was predominantly filled with spongy mineralized tissue formations. A thin layer of hyaline cartilage (green) between the bone ends had not yet been mineralized. In the ABC group almost only fibrous tissue was found, completely lacking any cartilage or bone formation; (ABC: autologous blood clot group; CD133(+): CD133 cell treated group); cortical bone/yellow; bone marrow/dark red; cartilage/green; muscle/red [21]. (**B**) Photomicrographs of immune histological sections 6 weeks post-surgery of the CD133(+) (left) and the ABC (right) group (factor VIII staining); In the CD133(+) group quite dense vessel formations (red) were observed, especially in the endostal region at the zone where the hyaline cartilage formations are mineralized. (ABC: autologous clot group; CD133: CD133+ cell treated group) (Scale bar: 1 mm).

In the CD133(+) group the gap region was filled with mineralized tissue 6 weeks after osteotomy in all investigated animals. Predominantly woven bone formations sending out thin spurs bridging the gap were identified (Fig. 5A).

In the ABC group the gap was mainly filled with fibrous tissue. No bridging of the osteotomy gap with cartilage or mineralized tissues was observed. Neither in the periosteal nor in the endosteal region osteogenic clusters could be seen (Fig. 5A).

### Improved healing in CD133(+) group correlates with increased vessel density

In the CD133+ group the three-fold number of capillaries and blood vessels was formed within our ROI when compared with the ABC group (74 vessels/ROI vs. 23 vessels/ROI, p<0.001). The vessels were distributed evenly within the periosteal regions and the gap region. Within the gap region vessels were mostly found at the boundary between woven bone and cartilage (Fig. 5B).

In the ABC group we found scarce numbers of capillaries and blood vessels (Fig. 5B). The vessels were distributed almost exclusively within the periosteal regions and very scarce within the gap region and the fibrous tissue.

## Discussion

The presented study shows the potential of CD133(+) peripheral blood derived cells to strongly enhance angiogenesis *in vitro*. In correlation with these *in vitro* results an increased revascularization of the callus area was seen in the *in* vivo experiment after implementation of CD133(+) cells. This pro-angiogenic potential offers an explanation for the increased vascularisation and, possibly for the increased callus formation observed in the animal model. Thus, a local application of pro-angiogenic peripheral blood derived cells and specifically CD133(+) cells at the time of injury improved the bone healing outcome.

It is known that the revascularisation of injured tissues is a prerequisite for healing and the essential phase in the early progression of bone regeneration [32], [33]. CD34(+), and CD133(+) cells have been previously described to bare angiogenic properties [12]–[14], [16], [17].

Even though CD34(+) hematopoietic stem cells are already considered as a promising candidate to further angiogenesis during bone regeneration [13], cells positive for the CD133 marker showed the highest advantage over the respective negative cells subset in our studies. CD133(+) cells have been introduced as a possible cell therapeutic for the treatment of ischemic conditions [34], but so far these cells have not been considered as a new therapy option in the context of bone injury.

By comparing both cell types in the context of bone regeneration in an model of biological impaired fracture healing only the CD133(+) cells showed a significant impact on fracture repair. That fact correlated with an increased angiogenic capacity in our *in vitro* analysis and an elevated gene expression of b-FGF and PDGFa. The high expression of these growth factors might be responsible for the healing outcome observed, as it is known that the establishment of a non-union is correlated with a lowered serum concentration of circulating b-FGF as well as PDGF-AB [35]. Therefore in bone healing high concentrations of b-FGF and PDGF-AB are crucial. Beside its pro-angiogenic properties [36] b-FGF promotes also the proliferation of osteoblasts *in vitro* [37] and regulates bone formation *in vivo* [38]. PDGF is known to have an impact on angiogenesis [39] and plays a crucial role in bone regeneration [40]. Additionally, locally administered PDGF was demonstrated to improve bone healing in an rabbit osteotomy model [41]. Transplantation of the b-FGF and PDGFa expressing CD133(+) cells to the fracture gap might hence increase locally the concentration of these growth factors and lead thereby to a better revascularization and bone formation at the site of injury. Results of the study confirmed that the local application of CD133(+) cells increased vessel formation as distinctly higher numbers of vessels could be detected at the border between cartilage and woven bone in the osteotomy gap. This was coupled with a better bridging and mineralization of the femoral gap. Thus the angiogenic potential and the positive effect on bone healing of CD133(+) cells gained from peripheral blood was demonstrated. The lower regeneration capacity seen in animals treated with CD34(+) cells could be explained in this context by the lowered b-FGF and PDGFa expression that was found in comparison to CD133(+) cells.

Another important aspect of the therapeutical concept to further bone healing by CD133(+) cell transplantation leading to a better revascularisation of the injured region is the theory that osteogenic progenitors reach the fractured areas via vessels [42], [43]. The endochondral ossification ultimately relies on osteoblast and their progenitors for the synthesis and mineralization of bone matrix. As a first step to regain stability in the broken bone, cartilage is formed in the osteotomy gap by chondrocytes. Later on these chondrocytes undergo hyperthrophy indicating the progression to woven bone. At the interface of hypertrophic chondrocytes and woven bone osteoblasts appear and generate bone [43]. We observed in our studies at these interface region the highest amount of new blood vessels in the CD133(+) treated animals. Hence it can be assumed that osteoblast reached this area by the new formed blood vessels. The initial treatment with angiogenic precursor cells therefore might not only influence the initial revascularisation steps but also those occurring during the later phases of the healing cascade thus enabling a successful bone healing outcome. Beside an increased migration of cells, the forced revascularisation after transplantation of CD133(+) lead also to a better nutrition and oxygen supply of local cells found in the hematoma and the fracture callus and support their proliferation and differentiation.

Angiogenic precursors, as CD133(+) cells, can be isolated from the bone marrow or from peripheral blood. The bone marrow is a rich source of progenitor cells; however the limited available amount and high donor site morbidity reported by patients contradict this approach. Peripheral blood in contrast is an easy available cell source without donor site morbidity, which makes it attractive for cell therapeutic approaches. It is known, that progenitor cells, as CD34(+) or CD133(+) are detected with lower percentages in peripheral blood when compared to bone marrow [44]. Interestingly, Eghbali-Fatourechietal et al. reported that the number of circulating osteogenic precursors were enriched in patients with a bone injury [45] by a mobilization from the bone marrow. Therefore the available number of CD133(+) cells in peripheral blood might also increase after fracture, which would facilitate the extraction of a suitable cell number. This would also avoid a potential necessary cell mobilization by the administration of growth factors, as this could result in negative side effects [46]. Hence, the easy availability of peripheral blood without donor site morbidity is a valuable argument for this source.

## Conclusions

Locally applied precursor cell subsets gained from peripheral blood showed a potential to improve impaired bone healing in a female, aged rat osteotomy model. Computer tomography and histology revealed an improved bone healing outcome with notably more blood vessels in the perichondreal region. The increase in revascularization could be one possible explanation for the enhanced healing outcome. This study demonstrates the suitability of CD133(+) cells from peripheral blood to further bone healing and gives an indication that peripheral blood is a promising source for progenitor cells circumventing the problems associated with bone marrow extraction.
